# Supplementary material for: Follistatin is a novel therapeutic target and biomarker in FLT3/ITD acute myeloid leukemia
Source: EMBO Mol Med. 2020 Mar 5;12(4):e10895. doi: 10.15252/emmm.201910895 (PMC7136967; doi:10.15252/emmm.201910895)
Supplement: Supplementary file 1 — Appendix [file EMMM-12-e10895-s001.pdf]

Appendix (Table of content):

1. Supplemental Figure S1 to S11 and Legends

2. Supplemental Table S1

Follistatin is a novel therapeutic target and biomarker in FLT3/ITD acute myeloid leukemia

Running Title: FST as biomarker and therapeutic target

Bai-Liang He,[1, 2, #] Ning Yang,[1, #] Cheuk Him Man,[1] Nelson Ka-Lam Ng,[1] Chae-Yin Cher,[1] Ho-Ching Leung,[1] Leo Lai-Hok Kan,[1] Bowie Yik-Ling Cheng,[1] Stephen Sze-Yuen Lam,[1] Michelle Lu-Lu Wang,[1] Chun-Xiao Zhang,[1] Hin Kwok,[3] Grace Cheng,[3] Rakesh Sharma,[3] Alvin Chun-Hang Ma,[4] Chi-Wai Eric So,[5] Yok-Lam Kwong,[1] and Anskar Yu-Hung Leung[1, *]

[1]Division of Hematology, Department of Medicine, Li Ka Shing Faculty of Medicine, The University of Hong Kong, 21 Sassoon Road, Pokfulam, Hong Kong SAR, China; [2]Guangdong Provincial Key Laboratory of Biomedical Imaging, The Fifth Affiliated Hospital, Sun Yat-sen University, Zhuhai, Guangdong Province 519000, China; [3]Centre for Genomic Sciences, 6th Floor, The Hong Kong Jackey Club Building for Interdisciplinary Research, The University of Hong Kong, 5 Sassoon Road, Pokfulam, Hong Kong SAR, China; [4]Department of Health Technology and Informatics, The Hong Kong Polytechnic University, Hung Hom, Kowloon, Hong Kong SAR, China; [5]Leukemia and Stem Cell Biology Group, Division of Cancer Studies, Department of Hematological Medicine, King's College London, Denmark Hill Campus, London SE5 9NU, UK. [#]Contributed equally to this work.

*Correspondence: **Anskar Yu-Hung Leung (M.D./Ph.D.)**

Address: Room K418, K Block, Queen Mary Hospital, Pokfulam, Hong Kong. Tel: (852)22553347; Fax: (852)29741165; Email: ayhleung@hku.hk

Figure S1

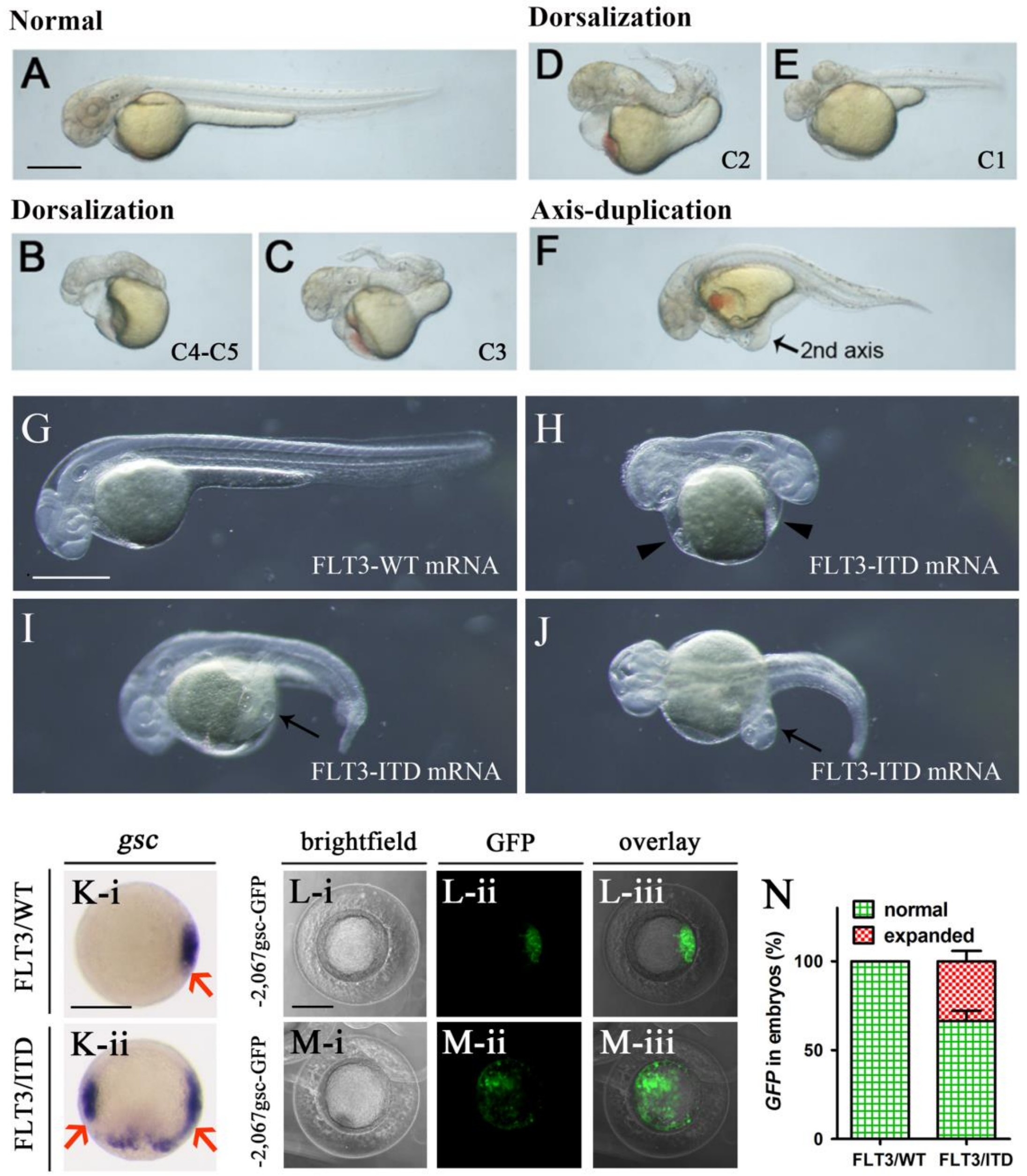

Figure S1. ***FLT3*/ITD induced axis-duplication and dorsalization in zebrafish embryos.**

A-F Definition of dorsalization (C5-C1) and axis-duplication phenotype in early zebrafish embryogenesis.

G-J The double-head and axis-duplication phenotype in *FLT3*/ITD mRNA-injected

zebrafish embryos at 2dpf. *FLT3*/WT mRNA-injected embryo was used as control. The arrows indicated the second head and the arrowheads indicated the duplicated hearts in the embryos.

K-N The expansion of Spemann's organizer after *FLT3*/ITD overexpression was detected by WISH of *gsc* (K, red arrows) and a -2,067gsc-GFP reporter assay (L and M) at 6 hpf. 50 pg plasmid vector, in which GFP expression is driven by zebrafish *gsc* promoter (-2,067), was co-injected with *FLT3*/WT or *FLT3*/ITD mRNA (150 pg) in zebrafish embryo at one-cell stage, respectively. The expression of GFP in the FLT3/WT- and FLT3/ITD-injected embryos (N) was used as a real-time *in vivo* surrogate marker for *gsc* expression and Spemann's organizer at 6 hpf.

Data information: Scale bar = 500 μm. All experiments were performed in triplicates.

Figure S2

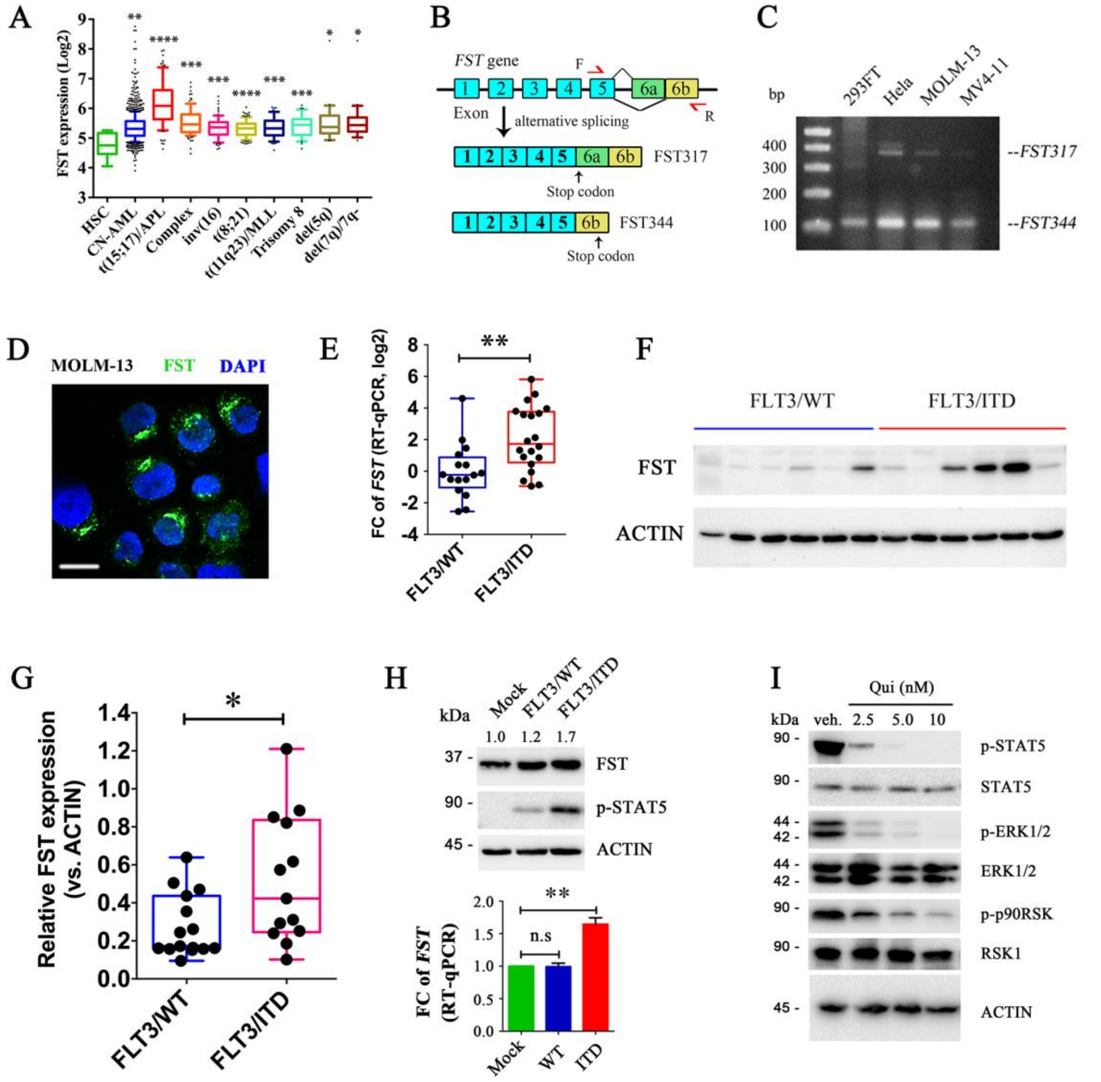

Figure S2. *FST* was upregulated in *FLT3*/ITD-mutated AML.

A *In-silico* analysis of *FST* expression in normal HSC and different subtypes of AML using BloodSpot program. The whiskers, boxes, and central lines represents the 10th-to-90th percentile, 25th-to-75th percentile, and the 50th percentile (median), respectively.

B-C Schematic representation of alternative splicing of *FST* gene (B) and RT-PCR of *FST317* and *FST344* expression in 293FT, Hela, MOLM-13 and MV4-11 cells (C).

D Detection of endogenous FST in MOLM-13 cells by immunofluorescence. Scale bar = 10 μm.

E-G FST expression was detected by RT-qPCR (E), and Western blotting (F and G) from *FLT3*/WT and *FLT3*/ITD AML patients (cytogenetic normal, leukemia blast > 80% at diagnosis). Panel F was the representative image showing FST expression from *FLT3*/WT and *FLT3*/ITD AML. β-ACTIN was used for normalization and quantification of FST expression in panel G. The whiskers, boxes, and central lines represents the minimum-to-maximum values, 25th-to-75th percentile, and the 50th percentile (median), respectively.

H Phosphorylation of STAT5 and FST expression were detected by Western Blotting and RT-qPCR in Hela cells-transfected with *FLT3*/ITD. The RT-qPCR experiment were performed in triplicates.

I FLT3 signaling were detected by Western blot in Ba/F3-*FLT3*/ITD cells-treated with Quizartinib (Qui in short) *in vitro* (0-10 nM) for 1 day.

Data information: In (E, G and H), data were presented as mean ± SEM. *P<0.05, **P<0.01 (Student's t-test), n.s: not significant.

Figure S3

A Upregulated genes in ML-2 (FST344 vs GFP)

GO term	p value	Genes
Signal transducer activity	0.01	FST, CSPG4, GJA1
Early endosome	0.02	RET, GJA1, C8ORF44-SGK3
MAPK cascade	0.03	RET, IL2RA, CCL5 *
Protein kinase activity	0.04	RET, CCL5, C8ORF44-SGK3
Cell proliferation	0.05	IL2RA, FSCN1, CSPG4

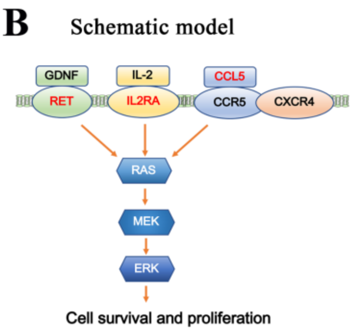

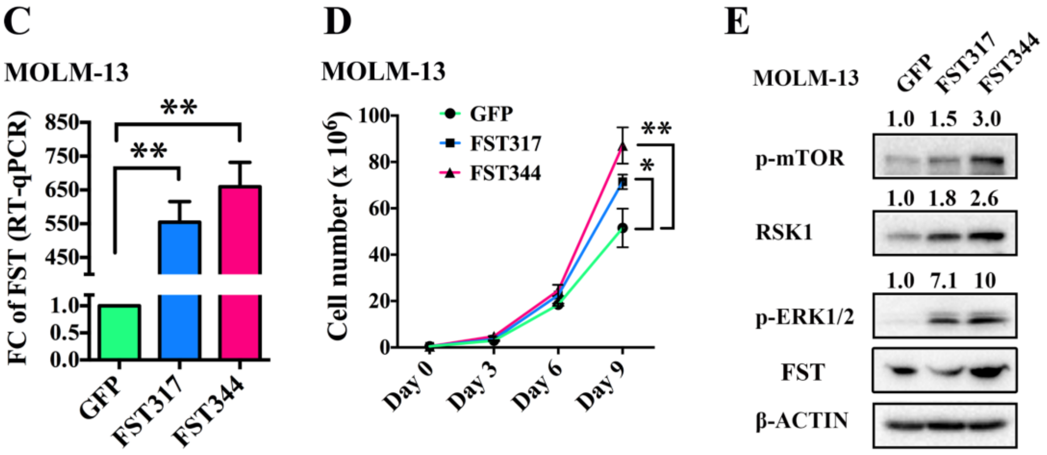

F Downregulated genes in ML-2 (FST344 vs GFP)

GO term	p value	Genes
Cell adhesion	5.15E-04	APP, CD36, DSG2, MYBPC3, VCAN, KIRREL2, THBS1, GPNMB, MYH10
Response to oxidative stress	0.01	APP, GAB1, PRDX2, SLC7A11
Differentiation	0.015	IGSF10, EPAS1, NAV1, SEMA4F, SYT3, SEMA3C, SPATA6, PPDPF
Immune response	0.028	TCF7, CD36, CNR2, PRG2, SEMA3C, THBS1
Negative regulation of FGF receptor signaling pathway	0.039	THBS1, GPC1
Extracellular matrix organization	0.045	APP, VCAN, THBS1, APBB2

Figure S3. Effect of *FST* overexpression on MOLM-13 and ML-2 cells.

A-B Gene ontology (GO) analysis of upregulated genes (from RNA-seq) in ML-2 cells-overexpressing *FST344* comparing to those of *GFP* overexpression (A). The upregulated genes *RET*, *IL2RA* and *CCL5* (asterisk) were enriched in the MAPK cascade which was shown in the schematic diagram in panel B.

C-E The effect of *FST* overexpression (C) on cell growth (D) and ERK activation (E) in *FLT3*/ITD MOLM-13 cells *in vitro*. The numbers above the blots indicated the fold change of p-mTOR, RSK1, and p-ERK1/2, respectively (GFP sample was used as control and set as 1). The RT-qPCR experiments were performed in triplicates.

F Gene ontology (GO) analysis of downregulated genes (from RNA-seq) in ML-2 cells-overexpressing *FST344* comparing to those of *GFP* overexpression.

Data information: In (C and D), data were presented as mean ± SEM. *P<0.05, **P<0.01 (Student's t-test).

Figure S4

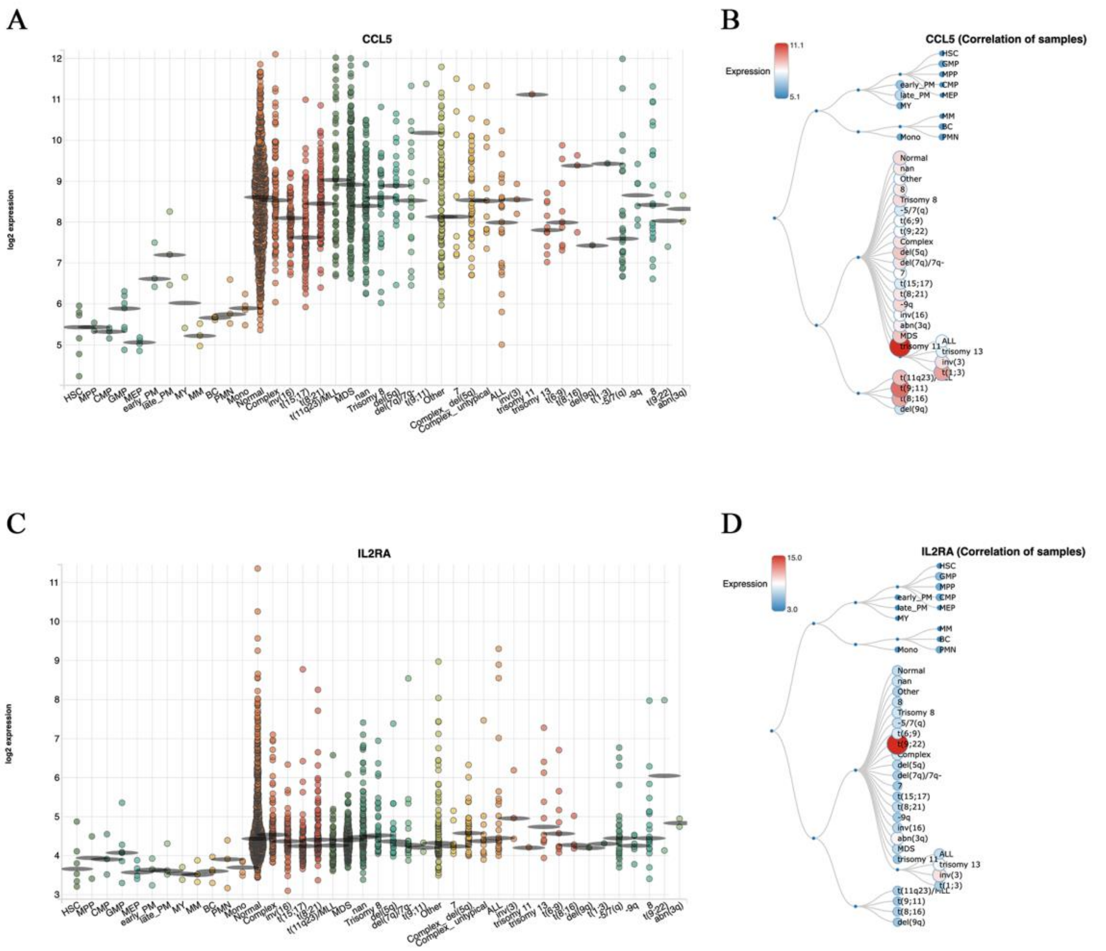

Figure S4. In silico analysis of CCL5 and IL2RA expression in normal hematopoietic tissues and human AML.

A-D The relative expression of CCL5 (A and B) and IL2RA (C and D) in normal hematopoietic tissues and human AML were analyzed by the public program Bloodspot (http://servers.binf.ku.dk/bloodspot/).

Figure S5

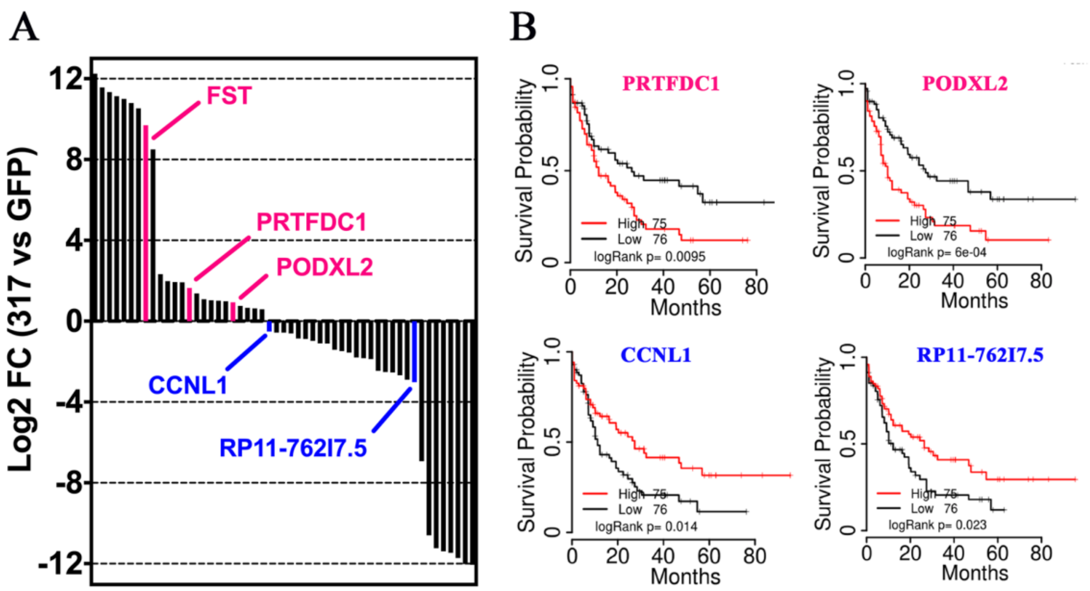

Figure S5. RNA-seq showing the differentially expressed gene after FST317 overexpression in ML-2.

A The differentially expressed genes in ML-2 cells (FST317 vs GFP) were shown as waterfall plot.

B The clinical relevance of these differentially expressed genes were analyzed based on the patients' survival data from TCGA-AML. Upregulation of *PRTFDC1* and *PODXL2*, and downregulation of *CCNL1* and *RP11-762I7.5* after *FST317* overexpression in ML-2 predicted the poor survival of AML patients.

Figure S6

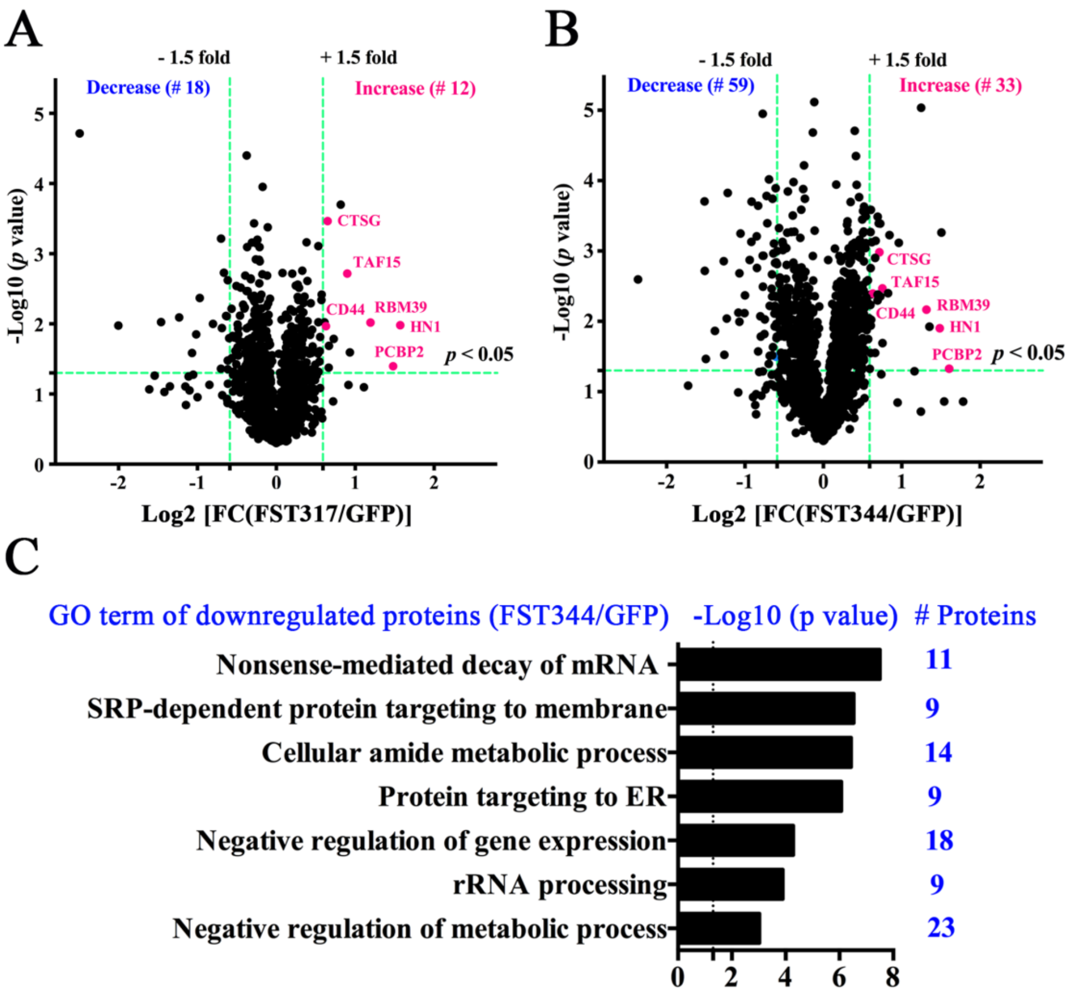

Figure S6. Proteomic analysis of ML-2 cells after *FST* overexpression.

A-B Volcano plot showing differentially expressed proteins after *FST317* and *FST344* overexpression in ML-2 comparing to those of *GFP* overexpression. CTSG, TAF15, CD44, RBM39, HN1, and PCBP1 were significantly upregulated in both *FST317* and *FST344*-overexpressing ML-2 cells.

C Gene ontology (GO) analysis of downregulated proteins in ML-2 cells-overexpressing *FST344* comparing to those of *GFP*. The downregulated proteins

associated with nonsense-mediated decay of mRNA were most significant.

Figure S7

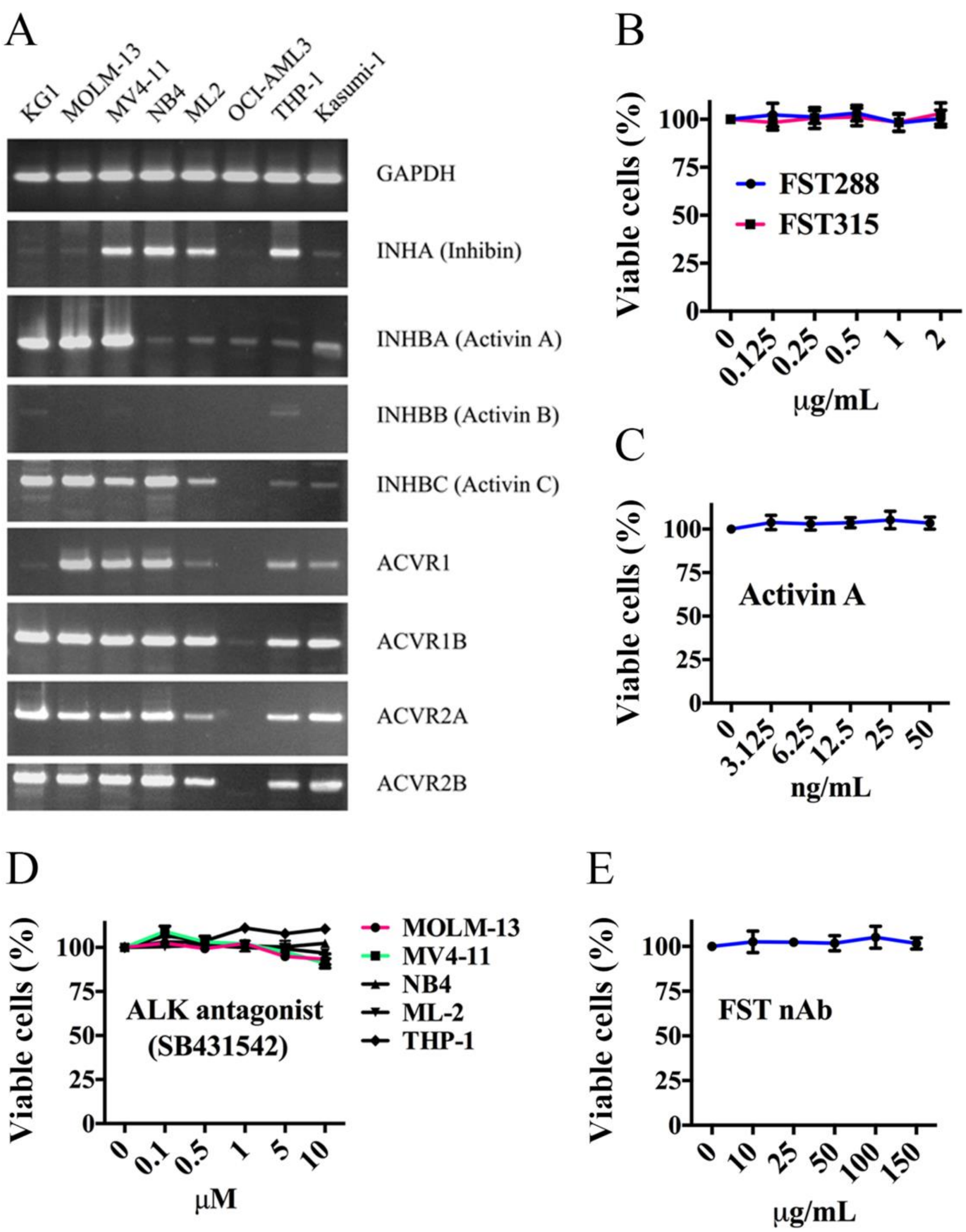

Figure S7. Exogenous FST and Activin treatment on AML cell growth.

A Gene expression of Activin and its receptors in AML cell lines by semi-quantitative PCR. *GAPDH* was used as house-keeping gene.

B-C The effect of exogenous FST and Activin A on MOLM-13 cell growth after 3 days treatment *in vitro*.

D The effect of Activin receptor antagonist (SB431542) on cell growth of different AML cell lines after 3 days treatment *in vitro*.

E The effect of FST neutralizing antibody on MOLM-13 cell growth after 3 days treatment *in vitro*.

Data information: In (B-E), the data were presented as mean ± SEM.

Figure S8

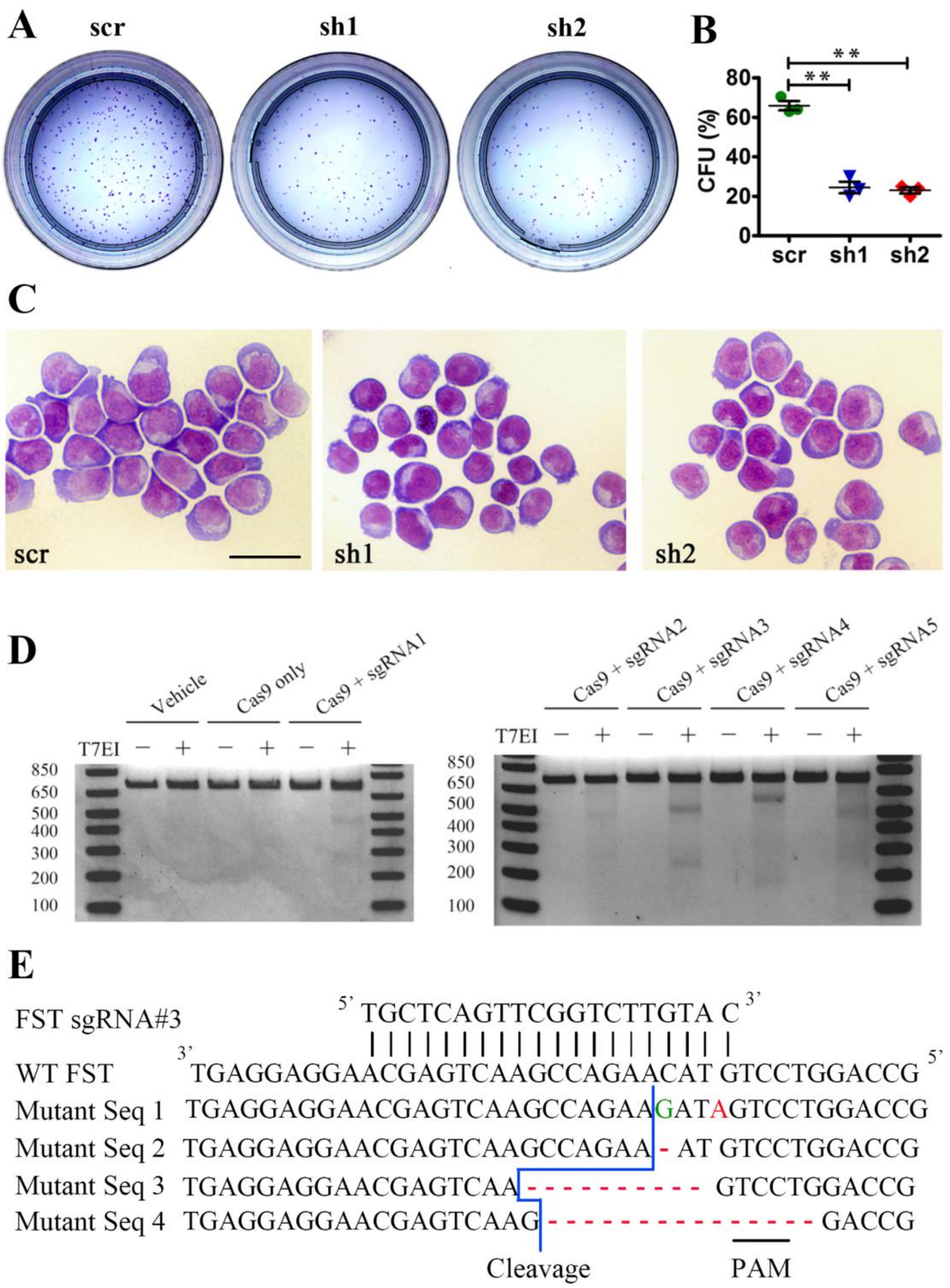

Figure S8. *FST* targeting by shRNA and CRISPR/Cas9 in *FLT3*/ITD AML cell lines.

A-C The clonogenicity (A and B) and morphology (C) of MV4-11 cells after *FST*

knockdown *in vitro*. Scale bar = 20 μm (C). In (B), the data were presented as mean ± SEM. **P<0.01 (Student's t-test)

D-E T7EI assay (D) and sanger sequencing (E, for sgRNA#3) detecting the genome editing efficiency of different sgRNAs for human *FST* in 293FT cells. Genomic DNA was extracted from 293FT cells at 3 days post transfection. Fragments containing the sgRNA target sites were PCR amplified, purified, denatured, annealed to form the heteroduplex, and digested with or w/o T7 Endonuclease I (T7EI) (D). To detect these insertion and deletion, fragments containing the sgRNA#3 target site were ligated into pGEM-T-easy vector for sanger sequencing (E). Green: mismatch; Red: insertion and deletion; PAM: protospacer adjacent motif.

Figure S9

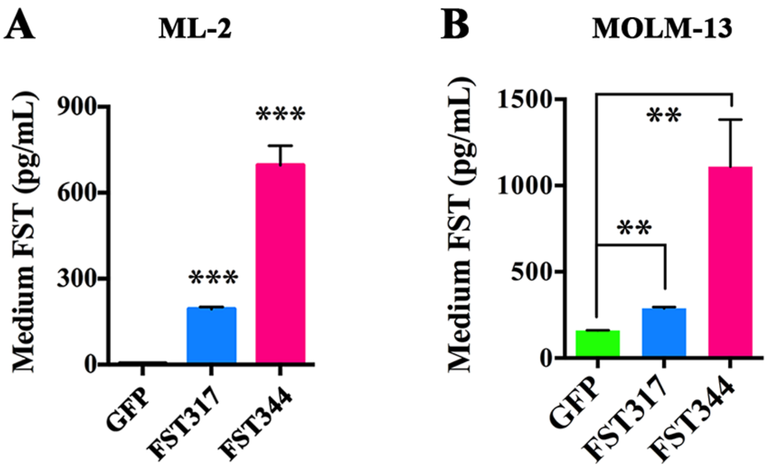

Figure S9. *FST* overexpression resulted in increased FST level in culture medium.

FST in the culture medium was measured by ELISA from ML-2 (A) and MOLM-13 (B) cells stably expressing *GFP*, *FST317*, and *FST344*, respectively. The ELISA experiments were performed in triplicates and the data were presented as mean ± SEM. **P<0.01, ***P<0.001 (Student's t-test)

Figure S10

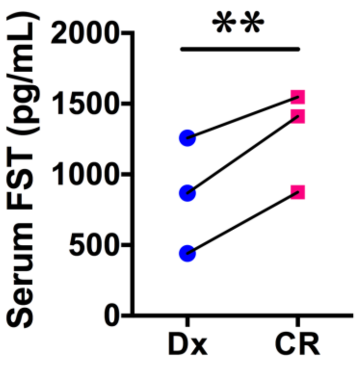

Figure S10. Serum FST level from FLT3/WT AML patients treated with conventional chemotherapy.

Serum FST levels were measured by ELISA from FLT3/WT AML patients treated with conventional “7+3” chemotherapy at diagnosis (Dx) and complete remission (CR). The data were presented as scatter dot plot. **P<0.01 (Student’s t-test).

Figure S11

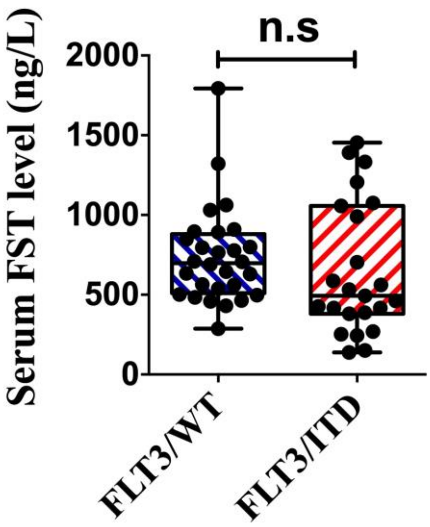

Figure S11. Serum FST levels from FLT3/WT and FLT3/ITD AML patients from our achieved samples.

Serum FST levels were measured by ELISA from FLT3/WT and FLT3/ITD AML patients at diagnosis. The data were presented in box plot. The whiskers, boxes, and central lines represented the minimum-to-maximum values, 25th-to-75th percentile, and the 50th percentile (median), respectively. n.s: not significant (Student's t-test).

Supplemental Table S1

Figure and panel	comparing groups	p-value	Symbol
Fig 1, panel K	DMSO vs Qui 5.0 μM (dorsalization)	1.51E-04	***
	DMSO vs Qui 5.0 μM (axis duplication)	1.15E-04	***
Fig 1, panel L	FLT3/WT vs FLT3/ITD (fst)	5.74E-03	**
	FLT3/WT vs FLT3/ITD (gsc)	7.95E-03	**
	FLT3/WT vs FLT3/ITD (fzd4)	3.23E-02	*
Fig 2, panel N	WT vs ITD	1.17E-04	***
Fig 2, panel O	WT vs ITD	9.23E-03	**
Fig 3, panel C	IgG vs p-CREB (c-Fos)	1.09E-04	***
	IgG vs p-CREB (FST)	3.28E-03	**
Fig 3, panel D	Red vs purple	5.99E-05	***
	Green vs purple	9.72E-04	***
Fig 3, panel G	Ba/F3-P vs Ba/F3-ITD (DMSO)	7.37E-05	***
	DMSO vs Qui-2.5 nM (Ba/F3-ITD)	9.54E-05	***
	DMSO vs Qui-5.0 nM (Ba/F3-ITD)	1.17E-05	***
	DMSO vs Qui-10 nM (Ba/F3-ITD)	1.11E-05	***
Fig 3, panel H	DMSO vs Qui-10 nM	2.42E-04	***
Fig 3, panel J	DMSO vs BRD7389 (Ba/F3-ITD)	8.76E-04	***
	DMSO vs BRD7389 (MOLM-13)	1.39E-03	**
	DMSO vs BRD7389 (MV4-11)	1.18E-03	**
Fig 4, panel B	Green vs blue	2.42E-03	**
	Green vs red	4.26E-03	**
Fig 4, panel C	Green vs blue	7.91E-03	**
	Green vs red	7.40E-03	**
Fig 4, panel E	Green vs blue	2.06E-02	*
	Green vs red	5.20E-03	**
Fig 4, panel G	Green vs red	2.90E-03	**
Fig 4, panel H	GFP vs FST317	3.40E-02	*
	GFP vs FST344	2.40E-03	**
Fig 4, panel J	GFP vs FST344 (RET)	8.33E-03	**
	GFP vs FST344 (IL2RA)	2.08E-02	*
	GFP vs FST344 (CCL5)	9.33E-04	***

Fig 5, panel C	scr vs sh1	8.42E-03	**
	scr vs sh2	3.42E-03	**
Fig 5, panel E	scr vs sh1	1.90E-02	*
	scr vs sh2	5.70E-03	**
Fig 5, panel G	Green vs blue	4.58E-02	*
	Green vs red	3.16E-02	*
Fig 5, panel H	scr vs sh1	5.34E-03	**
	scr vs sh2	3.29E-03	**
Fig 6, panel B	Cas9 vs sgRNA#3	6.22E-03	**
	Cas9 vs sgRNA#4	5.32E-03	**
Fig 6, panel D	Cas9 vs sgRNA#3	4.45E-02	*
	Cas9 vs sgRNA#4	2.89E-02	*
Fig 6, panel F	Neg-ASO vs FST-ASO3	7.13E-03	**
Fig 6, panel G	Neg-ASO vs FST-ASO3	9.41E-03	**
Fig 7, panel C	$Flt3^{+/+}$ vs $Flt3^{ITD/+}$	9.70E-03	**
Fig 7, panel D	$Flt3^{+/+}$ vs $Flt3^{ITD/+}$	9.80E-03	**
Fig 7, panel G	pre-injection vs week 2	1.55E-02	*
Fig 7, panel H	scr vs sh1	3.64E-02	*
	scr vs sh2	3.54E-02	*
Fig 7, panel K	Pre vs week 6	8.89E-03	**
Fig 7, panel N	Pre vs 1	6.72E-03	**
	Pre vs 2	3.74E-03	**
Fig S2, panel A	HSC vs CN-AML	2.70E-03	**
	HSC vs t(15;17)/APL	6.00E-05	****
	HSC vs Complex	1.00E-04	***
	HSC vs inv(16)	2.00E-04	***
	HSC vs t(8;21)	7.00E-05	****
	HSC vs t(11q23)/MLL	3.00E-04	***
	HSC vs Trisomy 8	5.00E-04	***
	HSC vs del(5q)	1.70E-02	*
	del(7q)/7q-	1.26E-02	*
Fig S2, panel E	FLT3/WT vs FLT3/ITD	2.60E-03	**
Fig S2, panel G	FLT3/WT vs FLT3/ITD	3.76E-02	*

Fig S2, panel H	Mock vs ITD	7.32E-03	**
Fig S3, panel C	GFP vs FST317	5.96E-03	**
	GFP vs FST344	4.78E-03	**
Fig S3, panel D	FST317 vs GFP (Day 9)	2.12E-02	*
	FST344 vs GFP (Day 9)	4.64E-03	**
Fig S8, panel B	scr vs sh1	5.22E-03	**
	scr vs sh2	3.96E-03	**
Fig S9, panel A	FST317 vs GFP	2.84E-04	***
	FST344 vs GFP	6.15E-04	***
Fig S9, panel B	FST317 vs GFP	2.04E-03	**
	FST344 vs GFP	9.25E-03	**
Fig S10	Dx vs CR	9.79E-03	**

Supplemental Table S1

The p-values in the main figures and Appendix were summarized. *P<0.05, **P<0.01, ***P<0.001.
