# Supplementary figures and images for: Follistatin is a novel therapeutic target and biomarker in FLT3/ITD acute myeloid leukemia
Source: EMBO Mol Med. 2020 Mar 5;12(4):e10895. doi: 10.15252/emmm.201910895 (PMC7136967; doi:10.15252/emmm.201910895)

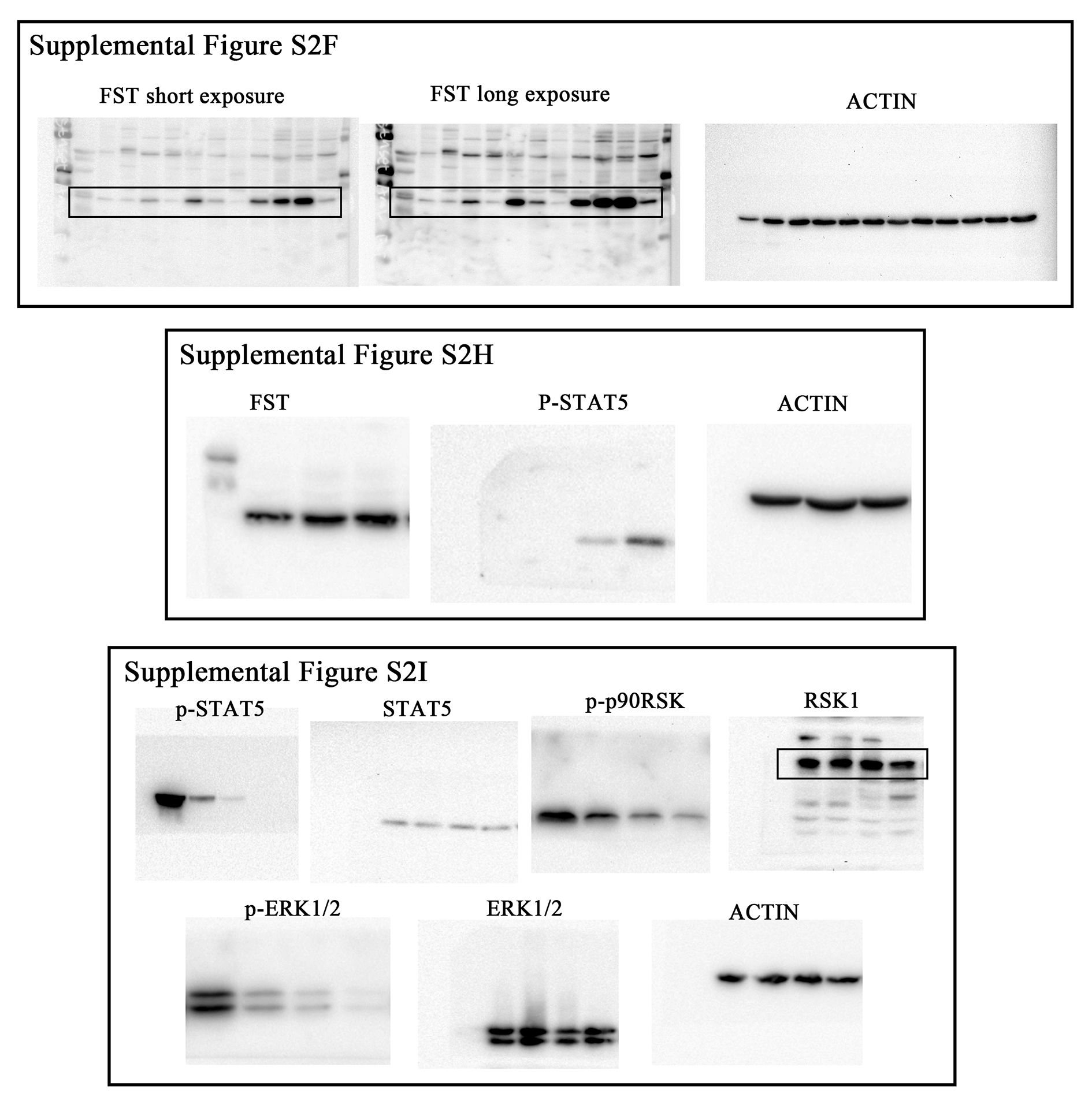

Supplement: Supplementary file 7 — Source Data for Appendix [file EMMM-12-e10895-s012.zip › EMM-2019-10895-V3_SourceDataForAppendixFigureS2_07Feb2020.tif]

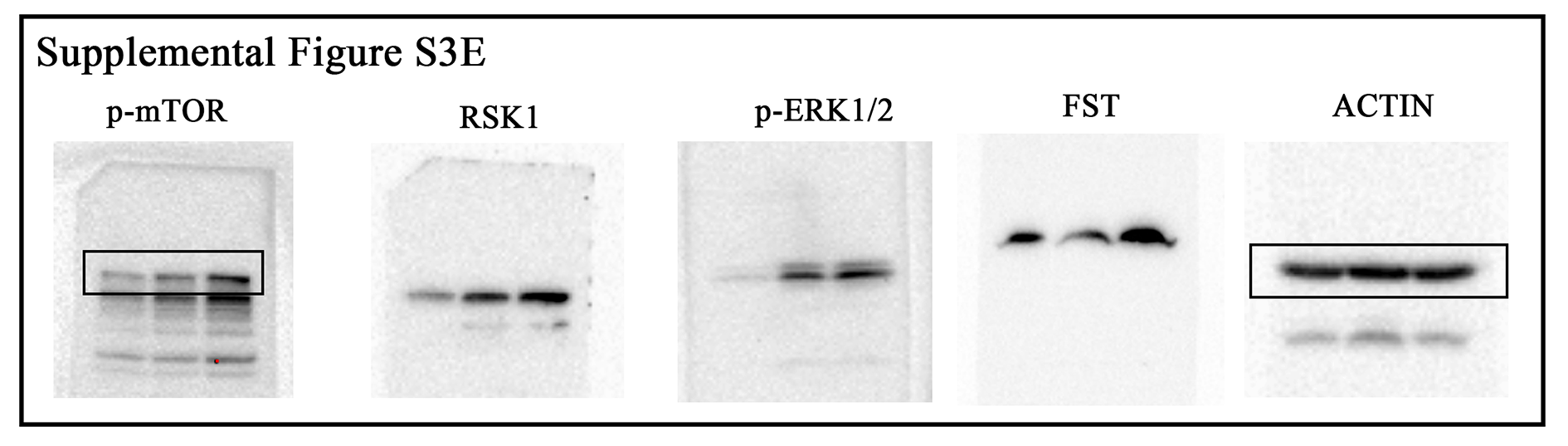

Supplement: Supplementary file 7 — Source Data for Appendix [file EMMM-12-e10895-s012.zip › EMM-2019-10895-V3_SourceDataForAppendixFigureS3E_07Feb2020.tif]

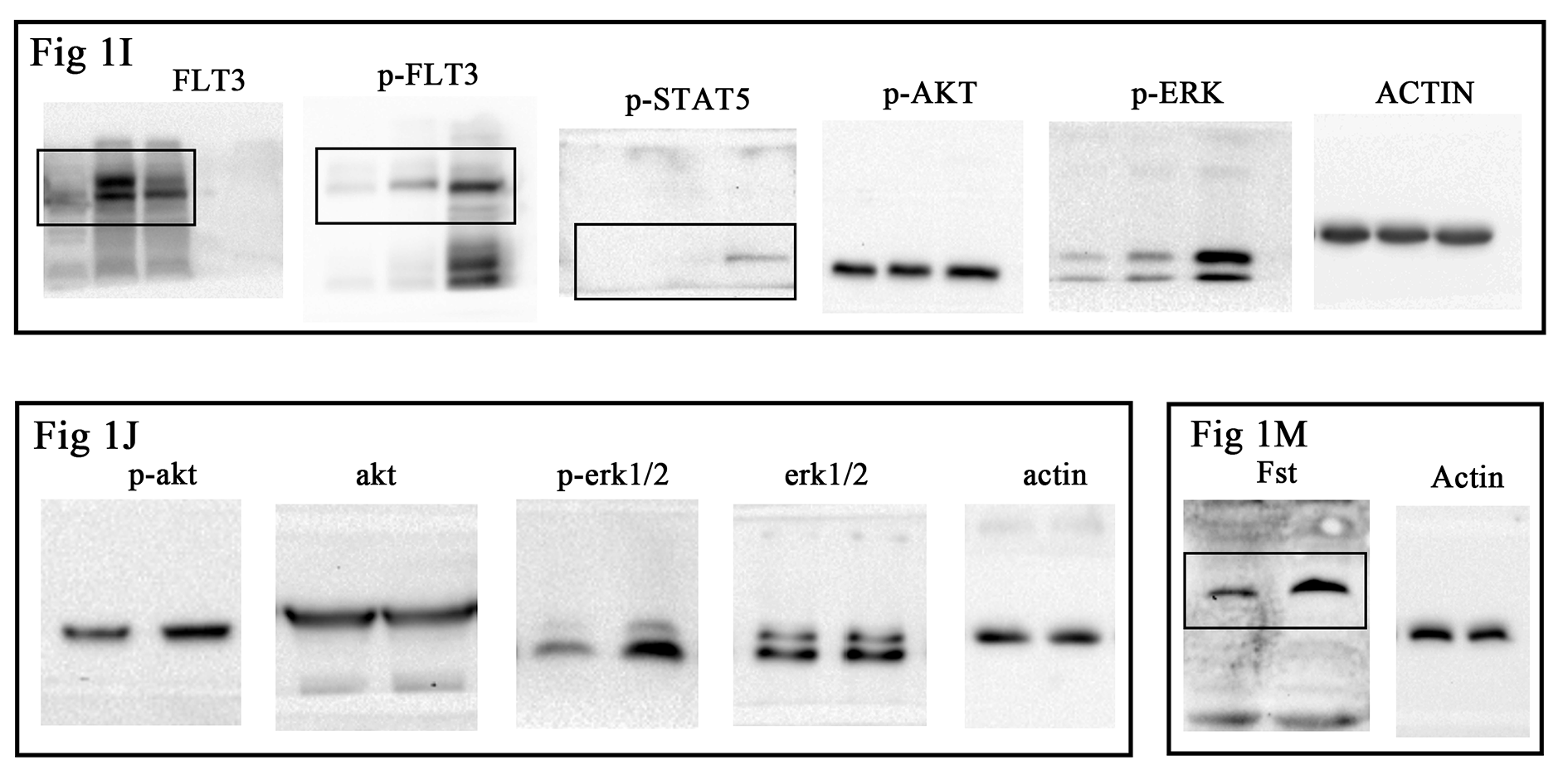

Supplement: Supplementary file 9 — Source Data for Figure 1 [file EMMM-12-e10895-s007.tif]

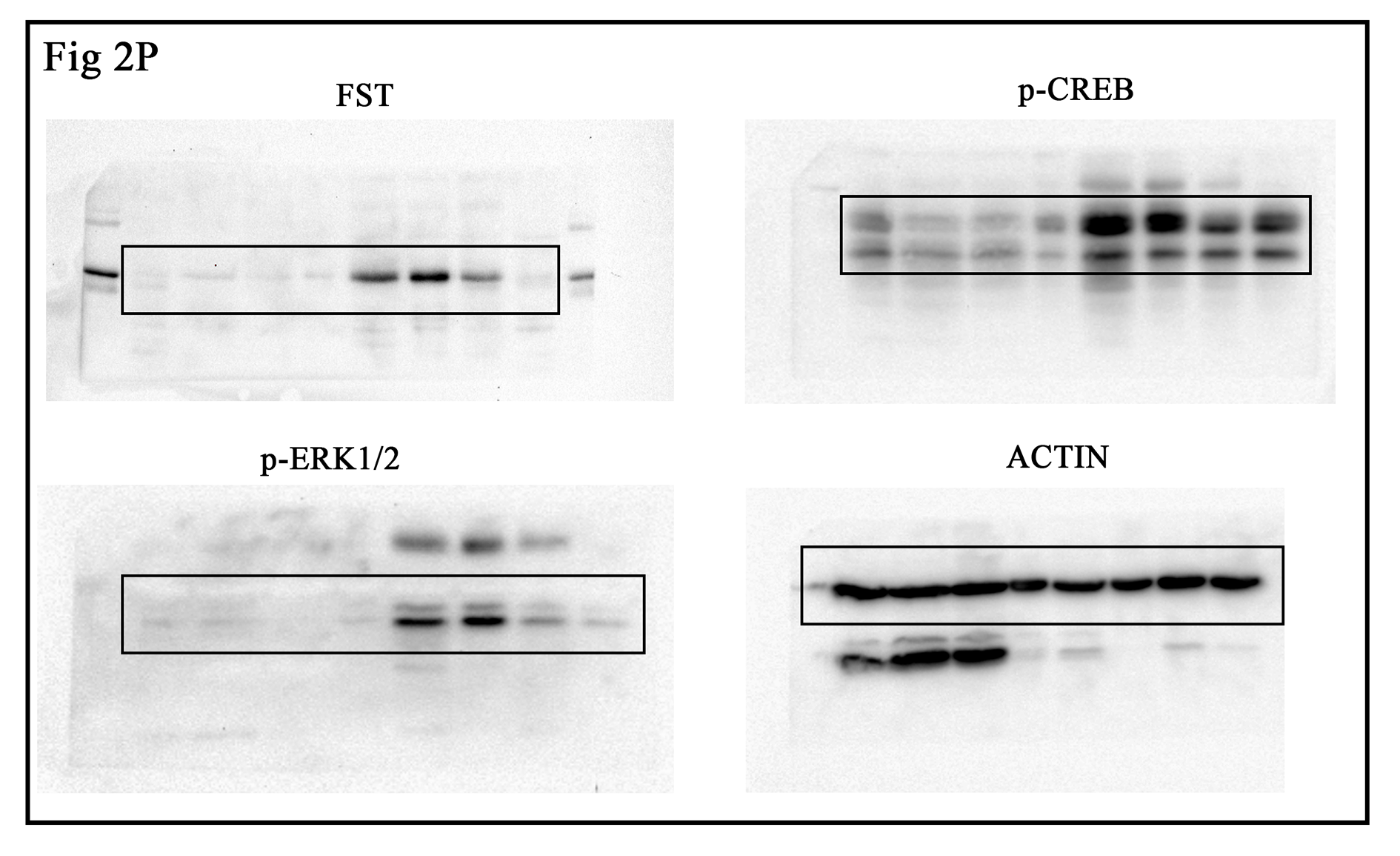

Supplement: Supplementary file 10 — Source Data for Figure 2 [file EMMM-12-e10895-s008.tif]

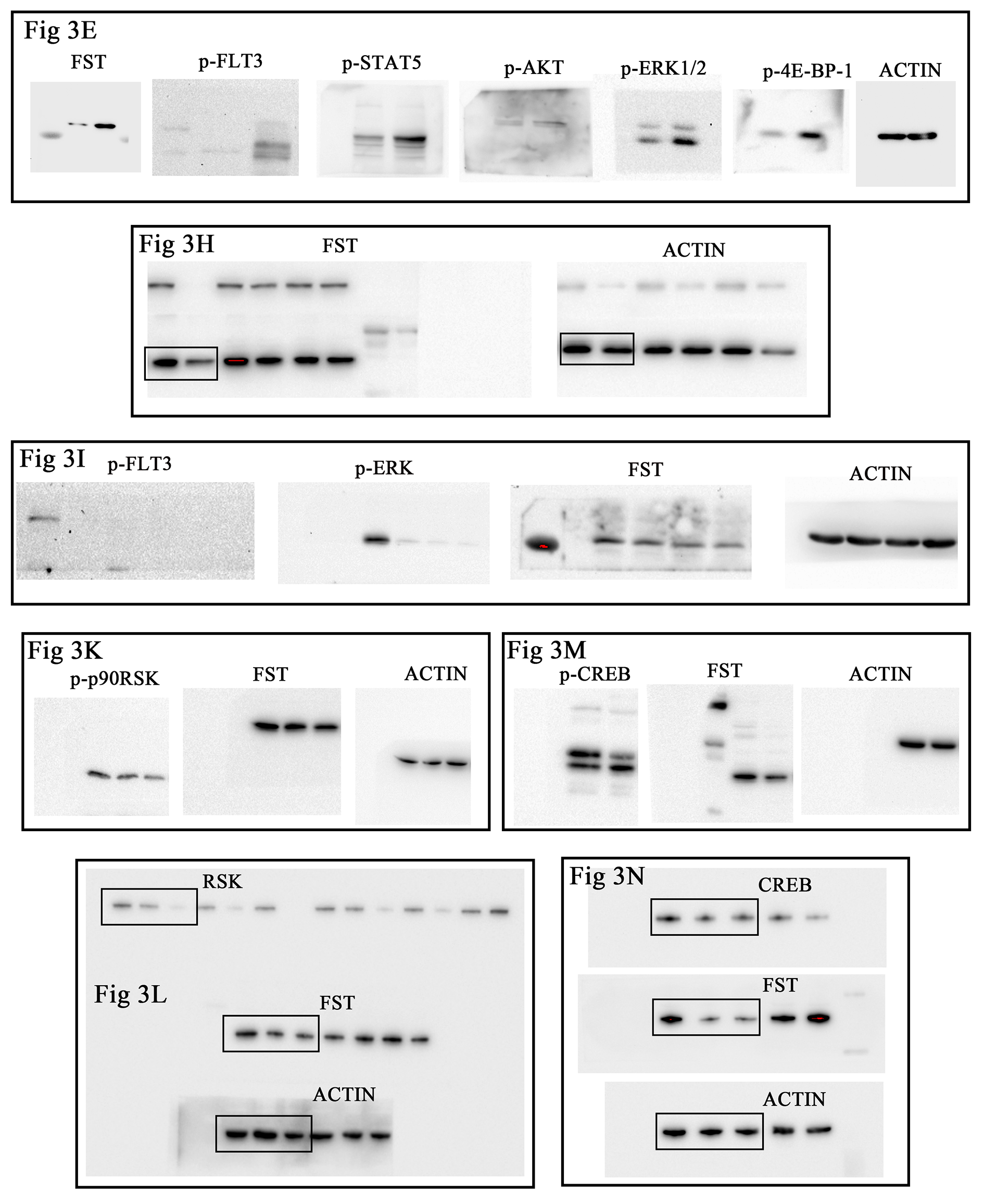

Supplement: Supplementary file 11 — Source Data for Figure 3 [file EMMM-12-e10895-s009.tif]

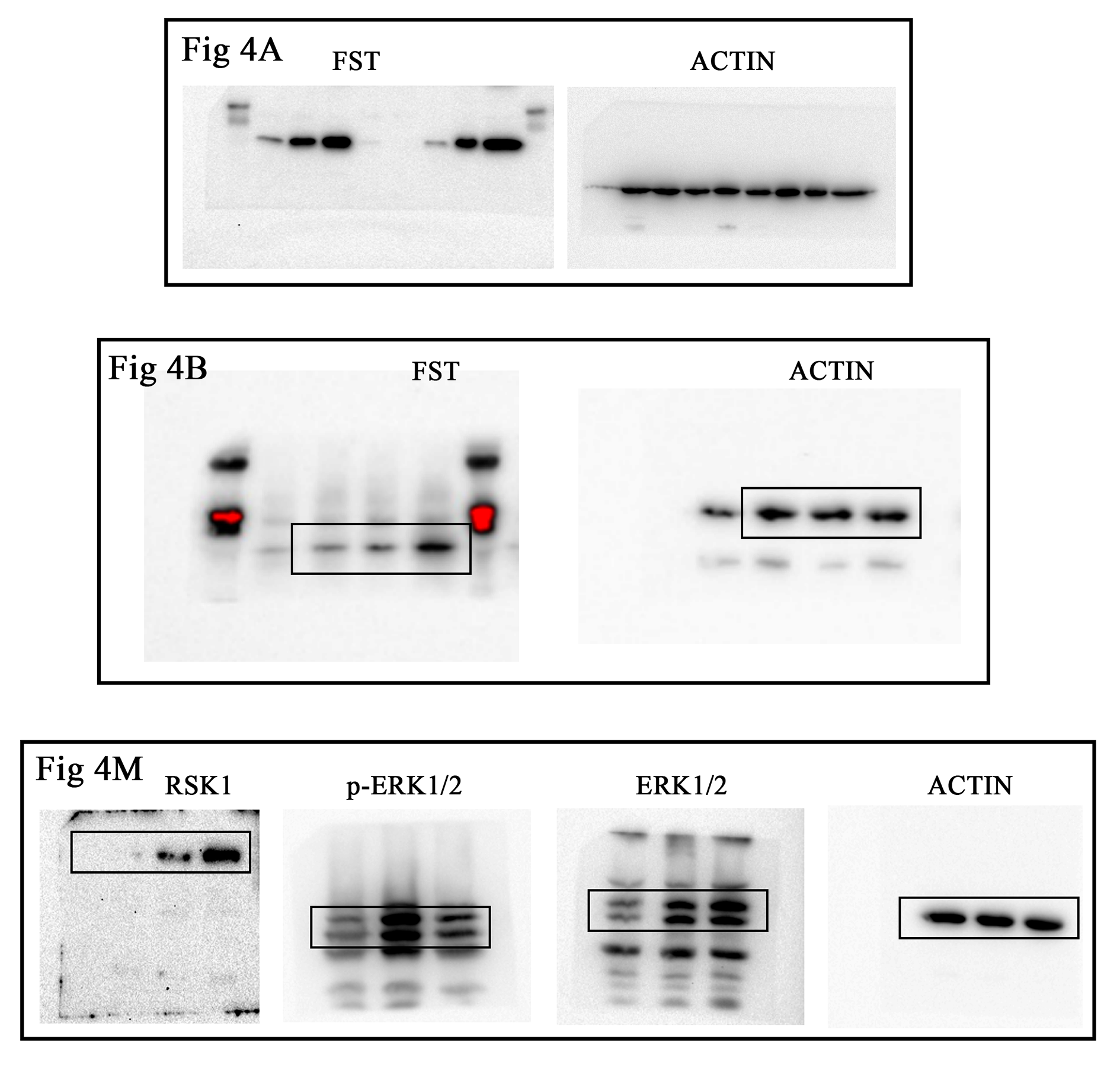

Supplement: Supplementary file 12 — Source Data for Figure 4 [file EMMM-12-e10895-s010.tif]

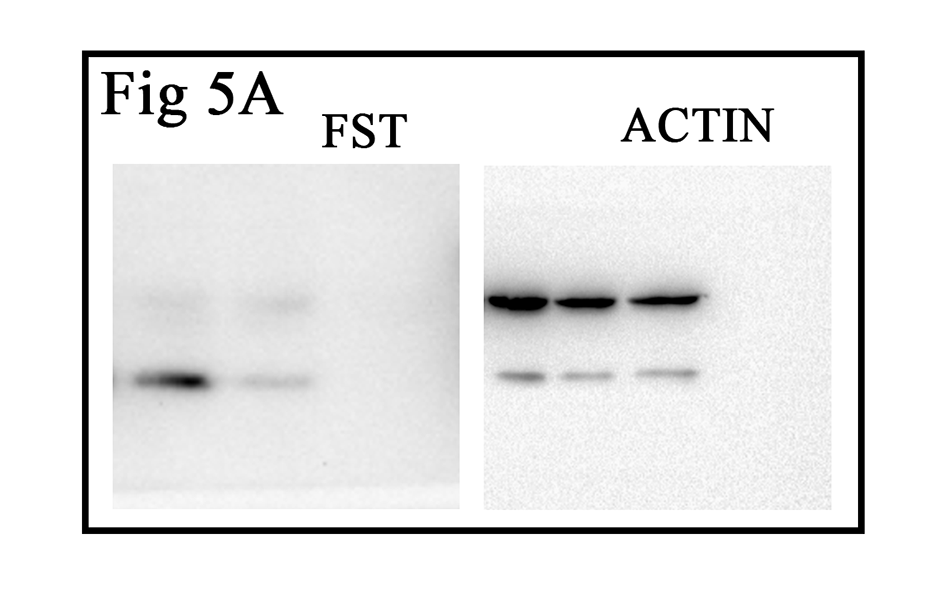

Supplement: Supplementary file 13 — Source Data for Figure 5 [file EMMM-12-e10895-s011.tif]
